# Exploratory comparison of Healthcare costs and benefits of the UK’s Covid-19 response with four European countries

**DOI:** 10.1093/eurpub/ckab019

**Published:** 2021-03-18

**Authors:** Howard Thom, Josephine Walker, Peter Vickerman, Will Hollingworth

**Affiliations:** Bristol Medical School: Population Health Sciences, University of Bristol, Bristol, UK

## Abstract

**Background:**

In responding to Covid-19, governments have tried to balance protecting health while minimizing gross domestic product (GDP) losses. We compare health-related net benefit (HRNB) and GDP losses associated with government responses of the UK, Ireland, Germany, Spain and Sweden from UK healthcare payer perspective.

**Methods:**

We compared observed cases, hospitalizations and deaths under ‘mitigation’ to modelled events under ‘no mitigation’ to 20 July 2020. We thus calculated healthcare costs, quality adjusted life years (QALYs), and HRNB at £20,000/QALY saved by each country. On per population (i.e. per capita) basis, we compared HRNB with forecast reductions in 2020 GDP growth (overall or compared with Sweden as minimal mitigation country) and qualitatively and quantitatively described government responses.

**Results:**

The UK saved 3.17 (0.32–3.65) million QALYs, £33 (8–38) billion healthcare costs and £1416 (220–1637) HRNB per capita at £20,000/QALY. Per capita, this is comparable to £1455 GDP loss using Sweden as comparator and offsets 46.1 (7.1–53.2)% of total £3075 GDP loss. Germany, Spain, and Sweden had greater HRNB per capita. These also offset a greater percentage of total GDP losses per capita. Ireland fared worst on both measures. Countries with more mask wearing, testing, and population susceptibility had better outcomes. Highest stringency responses did not appear to have best outcomes.

**Conclusions:**

Our exploratory analysis indicates the benefit of government Covid-19 responses may outweigh their economic costs. The extent that HRNB offset economic losses appears to relate to population characteristics, testing levels, and mask wearing, rather than response stringency.

## Introduction

Covid-19 has caused severe health and economic damage since its emergence in Wuhan, China at the end of 2019. As of 13 November 2020, the World Health Organization has reported over 50 million confirmed cases and 1.29 million deaths globally.^1^ In the UK, there have been over a million cases and over 60 000 deaths. The economic damage has been similarly dramatic with the International Monetary Fund (IMF) reducing its forecast gross domestic product (GDP) growth for 2020 in the UK from 1.4% in January to −9.8% in October.^2^^,^^3^ Similar reductions have been observed across Europe and the world. Health system responses (e.g. test and trace), government recommended or mandated social distancing measures and financial assistance have so far been the primary response.^4^

The economic case for stringent social distancing measures has been questioned due to their impact on the economy and livelihoods.^5^^,^^6^ A cost-benefit analysis of government responses to Covid-19 requires an infectious disease model to project the counterfactual of what would have happened in the absence of government intervention. The first wave of Covid-19 in Europe was largely complete by July 2020; complete data on observed outcomes are therefore available for comparison with modelled outcomes, and it is thus possible to compare the costs and benefits of Government responses to this first wave.^1^ The costs of social distancing measures are largely borne by society and the economy. A potential approach is to simply compare health-related net benefits (HRNB; health gains minus healthcare costs) to losses in GDP. However, this would underestimate the value of government intervention on social distancing as some GDP losses are due to Covid-19 ill-health, reductions in global trade and voluntary changes in behaviour rather than mandatory social distancing measures. Some studies have found that the much of the reduction is due to global and voluntary changes.^7^^,^^8^

Zala (2020) was an early UK modelling study aiming to assist policy makers but not making use of observed data. They found that even strategies causing a 10% reduction in national income are cost-effective at £50 000 per QALY.^9^ Lifetables and age-specific QALY norms were used to estimate that 8.8 QALYs were lost for each Covid-19 death, similar to estimates by Briggs (2020).^10^ Miles (2020) compared predictions of outcomes under no mitigation to observed outcomes up to June 2020, using an informal estimate of 5 QALYs lost per death.^6^ They compared the reductions in actual GDP growth forecasts to costs and QALYs saved by government response. They found that continuing the lockdown was only cost-effective at a willingness-to-pay per QALY between £220 000 and £3.7 million. 

Building on this earlier work, we aim to quantify the HRNB of Government interventions and compare them to the loss in GDP associated with social distancing measures in five western European countries over the first wave of Covid-19. We use a new model of cost and QALYs of Covid-19 cases, hospitalizations and deaths, with an estimate of QALYs lost per death. We compare observed outcomes to new forecasts under no mitigation using an adaptation of a published open-source Covid-19 model.^11^ Multiple forecasts are considered to explore uncertainty. HRNBs are compared per capita with published reductions in 2020 GDP growth forecasts both in total and using Sweden as a minimal mitigation comparator. Differences are presented alongside a description of social distancing and other measures, allowing us to illustrate characteristics of a successful response.

## Methods

The comparisons cover the period 1 January to 20 July 2020 corresponding to the first wave of Covid-19.^1^ The ‘mitigation’ strategy is based on observed disease outcomes while ‘no mitigation’ is based on modelled projections.

### Comparator countries and description of response

Comparator countries were selected as they were high income Western European healthcare systems with a range of responses and outcomes to Covid-19. Sweden had a less stringent response than the UK, Germany had lower case and death rates, Ireland took earlier and more stringent action and Spain had similar case and death rates.^1^^,^^12^ We reviewed each country’s response with reference to Wikipedia, news reports and government websites.^13^ Government responses were categorized under international travel, mass gatherings, pubs/restaurants, education, stay-at-home measures, financial assistance, mask requirements and testing. Timings were relative to the first confirmed Covid-19 case and death in each country. As numbers of cases and tests are likely correlated, we report tests per Covid-19 death. For additional comparison, we used the maximum and average, up to 20 July, University of Oxford Stringency Index for each country.^12^ This composite measure is based on nine response indicators including school closures, workplace closures and travel bans.

### Observed outcomes under ‘mitigation’

Government and news websites from each of the countries were searched for data on the numbers of Covid-19 cases, hospitalization and deaths that took place from 1 January to 20 July 2020. Covid-19-related deaths rather than excess deaths were used as these were available for all countries of interest.

### Modelling outcomes under ‘no mitigation’

We conducted a search for existing models on PUBMED, the Arxiv and medRxiv preprint servers. We selected the London School of Hygiene and Tropical Medicine Centre for the Mathematical Modelling of Infectious Diseases (CMMID) Covid-19 model (version 1) for projections in all countries of interest. This is an open-source age-structured deterministic mathematical model of SARS-CoV-2 transmission.^11^^,^^14^^,^^15^ This model was chosen because it included projections for all countries of interest, allowed common structural assumptions between countries and enabled tailoring of virus introduction dates and reproduction numbers ($R_{0}$). It accounts for differences in susceptibility and symptomatic rate by age, with children less susceptible to infection and more likely to be asymptomatic than adults.

We used results of meta-analysis studies and preprints published before 26 February 2020 to estimate the ‘no mitigation’ $R_{0}$ at 2.7 (95% credible interval 1.6–3.9).^14^ Our scenario A uses 2.7 while scenarios B and C assume${\;R}_{0}$ to be 1.6 and 3.9, respectively. This $R_{0}$ point estimate was sufficiently early to avoid the influence of public health interventions. Other modelling studies have used similar estimates of 2.6 (uncertainty range: 1.5–3.5) and 3.8, and there is limited evidence of variation between western European countries.^16–18^ Default values were used for other parameters. The model forecasts for each ${\;R}_{0}$ scenario in each country were then downloaded including cumulative cases, ICU bed days, non-ICU bed days and deaths from virus introduction to 20 July 2020. Bed days were converted to numbers of admissions using the duration of non-ICU and ICU stays assumed by CMMID. These values and further modelling details are in the Appendix.

### Calculating HRNB of mitigation

Our calculations include QALYs lost from Covid-19 cases, hospitalizations and deaths. We include only direct health impacts and not indirect impacts such as mental health effects of bereavement or social distancing. We used influenza quality of life decrements for hospitalized or non-hospitalized cases who survive as estimates are not yet available for Covid-19 (values in Appendix). We estimate QALYs lost per Covid-19 death using country-specific distributions of age at death, life expectancy and age-specific quality of life norms (details in Appendix).^10^ Our baseline estimate of QALYs lost assumes an SMR of 1.1 (see Appendix), but there is uncertainty about the prevalence of comorbidities in those dying from Covid-19 compared with the general population.^19^ We therefore consider a sensitivity analysis with a high SMR of 2.0.

### Health-related costs of treatment

We estimated hospitalization costs in 2020 UK £pounds sterling from a UK NHS perspective. We assumed no healthcare cost for community Covid-19 cases as prescription medications typically are low cost, and over the counter medications are not funded by the NHS. Using the figures for hospital stay costs, percentage requiring high dependency or ICU, average days ICU and cost per day of ICU gives an average cost of £4847 per hospitalized patient (details in Appendix). We did not include costs for testing or tracing as the level under ‘no mitigation’ is unknown, and under ‘mitigation’ was not reported by all countries.

### Calculating direct HRNB of mitigation

The total QALYs lost and healthcare costs were estimated for the observed ‘mitigation’ and modelled ‘no mitigation’ strategies. Incremental QALYs gained and healthcare savings from mitigation were calculated. A monetary incremental HRNB was calculated by multiplying incremental QALYs by the conventional UK willingness-to-pay threshold of £20 000/QALY and subtracting incremental costs.^20^ These are presented per capita using 2020 population estimates.^21^

### Economic impact

We used the October 2020 IMF World Economic Outlook projections for GDP growth in 2020 to give estimates of growth with the Covid-19 pandemic and compared these to the corresponding January 2020 pre-pandemic projections.^3^^,^^22^ January IMF estimates for Sweden and Ireland were not available so instead Organization for Economic Cooperation and Development (OECD) 2020 projections from 21 November 2019 were used.^23^ We calculated pound sterling value of GDP changes using IMF estimates of total GDP value in October 2019 and OECD 2019 purchasing power parities.^2^^,^^24^ Final results are reported per capita using 2020 population estimates.^21^

Only part of the observed GDP loss is a result of government-mandated social distancing measures, with the rest a consequence of global trade changes, voluntary behaviour changes and productivity loss due to ill health. We therefore present two scenarios. The first assumes that under no mitigation, the 2020 GDP reduction would have been the same as that observed in Sweden. Sweden might be considered to represent the ‘minimal mitigation’ policy that is politically acceptable in a western European democracy during a global pandemic rather than ‘no mitigation’. In the second, we estimate the percentage of observed GDP loss that was offset by net QALYs gained and healthcare savings.

## Results

### Description of country responses to Covid-19

Apart from Sweden, response measures and timings were similar across countries (full details in Appendix). Ireland banned gatherings 15–28 days earlier than other countries. All countries except Sweden ordered pubs and restaurants to shut but Ireland did so up to 40 days earlier. Spain, Germany and the UK closed schools at a similar timepoint, roughly 30 days after Ireland. Sweden recommended secondary schools and universities to move to distance learning but kept nurseries and schools open. The UK introduced stay-at-home orders almost 30 days later than Ireland and 12 days after Spain. Sweden had no stay-at-home order. All countries introduced wage support at similar timepoints. Requirements for mask wearing were eventually introduced for all countries except Sweden, but Spain and Germany moved much earlier than the UK and Ireland. Most countries did not introduce comprehensive tracing until the very end of the first wave. Testing levels per death were much higher in Germany, with 812.8 compared with 324.6 for Ireland with the second highest level. According to the stringency Index, the UK had lower maximum level restrictions than Ireland and Spain but higher than Germany and Sweden. Ireland had the highest maximum level of restrictions. Average restriction index was very similar between countries other than Sweden. Sweden had the least stringent measures (maximum 64.81 vs. next lowest 76.85 and average 38.79 vs. next lowest 45.41).

### Comparison of responses to Covid-19 on cases, hospitalizations and deaths

**Figure 1:**
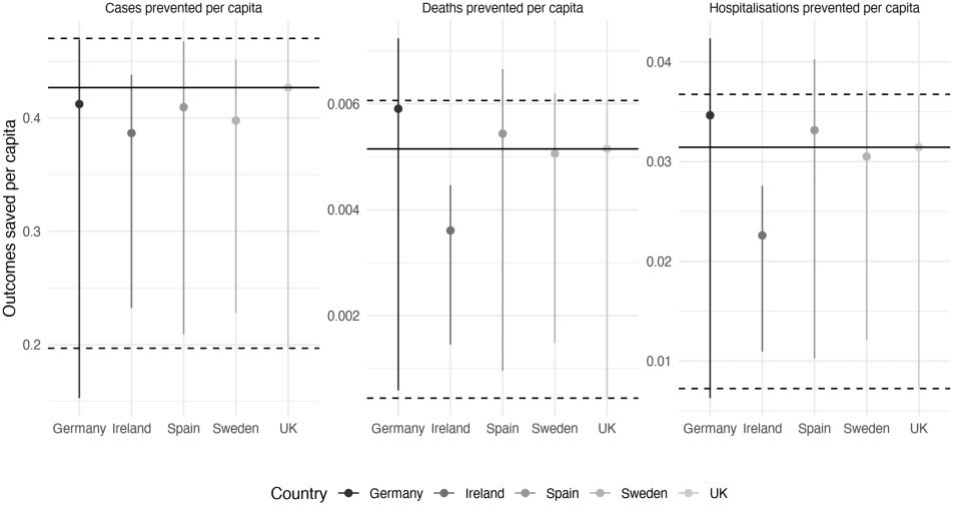
Outcomes saved per capita (PP). Central estimate is scenario A, whereas lower and upper limits correspond to scenarios B and C, respectively. Horizontal solid lines indicate UK estimates for ease of comparison. Values above these lines indicate greater benefit per capita. Comparison of cases is affected by levels of testing so focus should be on deaths and hospitalizations

Differences in prevented Covid-19 cases, hospitalizations and deaths under ‘mitigation’ are illustrated in Figure 1 , with full details in the Appendix.

Observed case numbers are affected by variance in levels of testing across countries limiting comparability. Hospitalizations and deaths per capita were highest in Sweden, Spain and the UK. Ireland and Germany had much lower hospitalizations and deaths.

Under base case scenario A ($R_{0}=2.7$), Spain and Germany prevented more hospitalizations and deaths per capita than the UK, whereas Sweden was similar to the UK. Ireland prevented fewer hospitalizations and deaths than all countries, likely driven by a younger population. Patterns are similar under scenario C ($R_{0}=3.9$) but reverse for Germany and Ireland under A ($R_{0}=1.6$); this nonintuitive reversal is due to a nonlinear relationship between $R_{0}$ and outcomes.

### Comparison of health-related costs, benefits and net benefits

The incremental health-related benefits (QALYs), costs, and per capita incremental net benefits are presented in Table 1. In scenario A ($R_{0}=2.7$), HRNBs per capita are highest for Germany, followed by Spain, UK, and Sweden. Ireland had almost 30% lower net benefit than Sweden. These findings are driven by prevented hospitalizations and deaths (Figure 1).

**Table 1:** Health-related benefits, costs and net benefits of the government response for SMR = 1.1 scenarios

Scenario	Country	Incremental benefit (millions of QALYs)	Incremental costs (£Billions)	INB per capita at £20 000/QALY (£)
A$R_{0}=2.7$	UK	3.166	−32.94	1416.6
	Ireland	0.162	−1.67	994.6
	Spain	2.297	−23.84	1492.6
	Germany	4.467	−43.22	1581.2
	Sweden	0.463	−4.78	1388.2
B All $R_{0}=1.6$	UK	0.319	−8.61	220.5
	Ireland	0.071	−0.82	453.3
	Spain	0.452	−8.00	364.6
	Germany	0.501	−8.03	215.3
	Sweden	0.149	−2.04	495.4
C All $R_{0}=3.9$	UK	3.649	−38.28	1637.4
	Ireland	0.197	−2.04	1206.6
	Spain	2.754	−28.75	1792.6
	Germany	5.357	−52.79	1907.6
	Sweden	0.555	−5.77	1668.1

Scenarios are for the ‘No mitigation’ simulations from the CMMID model which are compared with the observed outcomes. Comparisons up to 20 July 2020. (INB = Incremental health-related net benefit).

Under scenario B ($R_{0}=1.6$), Sweden and Ireland have the greatest health-related benefit, whereas the UK and Germany have the lowest, again driven by nonlinear modelling relationship between $R_{0}$ and outcomes (Figure 1). Under scenario C ($R_{0}=3.9$), almost the same pattern as the base case is found. Patterns are the same under SMR = 2.0, but HRNBs are marginally lower (see Appendix). Deaths account for 60–70% of incremental HRNBs, with the remainder due to hospitalizations (Appendix). Over 95% of the benefit of prevented hospitalizations is due to costs rather than QALYs.

Across all scenarios, with $R_{0}=2.7$ as base case, the UK response is estimated to have saved 3.17 million (ranging from 0.32 to 3.65 million) QALYs, £33 billion (£8–38 billion) in healthcare costs and gained £1416 (220–1637) per capita HRNB at £20 000/QALY.

### Comparison of economic impact of responses to Covid-19

Estimates of the economic impact of government responses are presented in Table 2. Spain and the UK had the worst GDP loss, Germany and Ireland had much smaller reductions in GDP. Despite high hospitalization and deaths, Sweden had the lowest reduction in GDP, possibly explained by lower severity restrictions.

**Table 2:** International comparison of GDP growth forecasts, losses due to the pandemic and government response, and % offset by health-related net benefit

Country	Population size	GDP in 2019 (£billion)	GDP per capita (£)	IMF Growth January 2020 (%)	IMF Growth October 2020 (%)	Reduction in GDP compared with no mitigation PP (£)		
						Scenario: Sweden GDP loss as ‘no mitigation’	Scenario: All GDP reduction due to response	
							GDP loss per capita (£)	% offset by health-related net benefit at £20k/QALY
UK	67 950 117	1865.64	27 456	1.4	−9.8	1455.17	3075.072	46.1 (7.17, 53.2)
Ireland	4 947 782	261.76	52 904	3.3^b^	−3	211.62	3332.974	29.8 (13.6, 36.2)
Spain	46 758 012	950.55	20 329	1.6	−12.8	1727.98	2927.401	51.0 (12.5, 61.2)
Germany	83 831 967	2627.07	31 337	1.1	−6	376.05	2224.954	71.1 (9.68, 85.7)
Sweden	10 110 601	359.67	35 574	1.2^b^	−4.7	0.00	2098.85	66.1 (23.6, 79.5)

a GDP growth is forecasted for 2020 in January (before pandemic) and October (after pandemic). One scenario assumes the growth loss of Sweden (i.e. 1.2 before pandemic and −4.7 after pandemic, giving reduction of 5.9%) represents growth that would be lost under no mitigation. Figures are per capita in pounds sterling.

b Ireland and Sweden based on OECD estimates from 21 November 2019.^23^

In scenario A, observed GDP loss exceeded HRNBs in all countries (Tables 1 and 2). The extent to which GDP losses were ‘offset’ by net health benefits ranged widely from a low of 30% in Ireland to 71% in Germany at the £20 000/QALY threshold (Table 2). These findings were very sensitive to assumptions about $R_{0}$. For example, in the UK, the extent to which GDP loss is offset was 46% in scenario A but ranged from 7 to 53% (Table 2). If, however, the 5.9% GDP loss observed in Sweden is considered the minimal level for European economies, given the effects of global trade and voluntary behavioural changes, then under scenario A, the net health benefits in Germany and Ireland exceed the GDP loss, whereas they fall short but are comparable in the UK and Spain. The analysis with SMR = 2.0 (Appendix) reduces net benefits per capita but does not change our overall findings.

**Figure 2:**
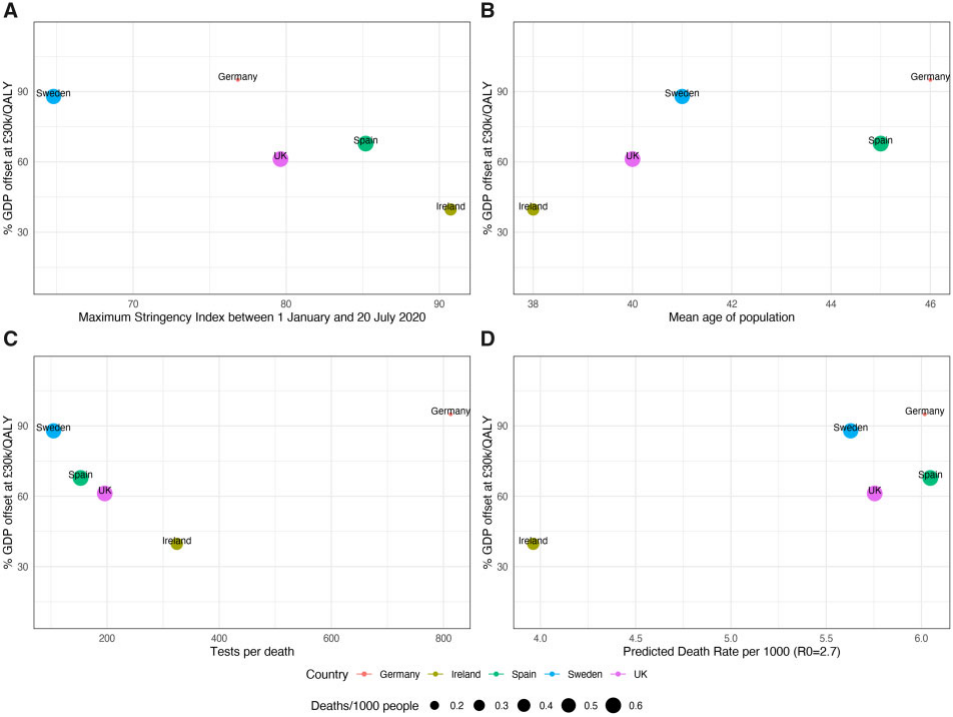
Comparison of % GDP loss offset by net health benefits at £20 000/QALY across countries and comparison with explanatory factors: (A) maximum stringency index, (B) median age of population,^21^ (C) tests per death and (D) predicted death rate under no mitigation scenario

Figure 2 illustrates that in countries (Ireland, Spain and the UK) with higher social distancing stringency, net health benefit did not offset a greater proportion of GDP loss. It illustrates that in Germany, with highest testing per death, net health benefits offset the greatest % GDP loss. It illustrates that in Ireland, with lowest predicted ‘no mitigation’ death rate, net health benefits of mitigation offset the lowest % GDP loss. It also suggests that countries with lower median age (e.g. Ireland) had lower offsets than those with higher age (e.g. Germany). None of these patterns are consistent, and there is insufficient data for statistical testing.

## Discussion

We have provided an exploratory comparison of health-related benefits and costs saved by government measures across European countries. Mitigation saved lives and reduced healthcare treatment costs substantially, with the HRNB in the UK being £1416 (220–1637) per capita. In all countries, projected GDP loss is higher than HRNBs, but this ignores that GDP would have reduced in the absence of government mandated social distancing measures. In the UK, 46.1 (7.1–53.2)% of GDP loss was offset by HRNB, but higher offset percentages were estimated in Germany (71.1%), Spain (51.0%) and Sweden (66.1%). Countries with tighter social distancing restrictions do not appear to perform better on this measure. Instead, the distinguishing features of Germany (best performing country) are higher testing, earlier mask wearing and higher median age. Germany having the highest % GDP offset may also be explained by having lowest absolute death and hospitalization rates.^7^ The defining feature of Ireland (worst performing country) is low likelihood of Covid-19 hospitalization and death, possibly explained by lowest median age (38 years).^25^ High % offset countries Germany and Spain may simply have had older populations with more to benefit (Figure 2). Sweden had lower levels of testing, no requirement for mask wearing and mostly voluntary but had similar health-related benefits to the UK; median age (41 years) is similar to the UK (40 years) again suggesting population susceptibility is a key determinant of success.

There are significant limitations to our analysis. There is limited understanding of plausible parameter ranges (e.g. $R_{0}$) and limitations of using an existing model so we could not conduct probabilistic sensitivity analysis. Costs of hospitalization were assumed to be independent of the number of hospitalizations; however, once capacity is breached, additional facilities would need to be constructed at substantial cost. We did not model testing or tracing costs. The UK has allocated £22 billion or £338 per capita on testing and tracing over 2020–2021 so this could become important for future evaluations.^26^ We did not model indirect health impacts such as the impact of either social distancing or a more severe pandemic on mental health or delivery of healthcare. There are limitations in the extent to which any single measure of stringency can capture complex Government policies. For example, Finland had a maximum of 67.6 and average of 36.6 compared with 64.8 and 38.8 in Sweden, respectively, despite introducing a strict lockdown.^12^ The CMMID model represents a worst case scenario for ‘no mitigation’ as it does not include voluntary changes in behaviour. Finally, our restriction to the first wave of the pandemic means that we may be comparing cases, hospitalizations or deaths postponed rather than prevented, and we take no account for possible herd immunity.

Estimating only the impact of government social distancing measures on GDP is difficult. Baker (2020) estimated that at least half of economic impact is due to global uncertainty, whereas Chen (2020) found that outbreaks and voluntary mobility reductions have a much greater impact.^8^^,^^27^ Sweden represents ‘minimal mitigation’ rather than ‘no mitigation’. Furthermore, Sweden’s neighbouring countries introduced greater restrictions and also achieved lower forecast reductions in GDP (e.g. Norway with maximum stringency index 79.6 and forecast −4.0% growth, Denmark with maximum index 72.2 and −4.5% growth).^12^^,^^22^ It is clear that there is no simple trade-off between economic pain and health gains. Finally, comparisons for Ireland may be skewed as its GDP per capita is subject to disproportionate globalization effects.^28^

A challenge to our % GDP loss offset is that the £20 000/QALY threshold relates to NHS expenditure, not society. Societal thresholds may range from £10 000/QALY to £70 000/QALY.^9^ The NHS uses a threshold of £50 000/QALY for patients at end-of-life, possibly relevant to patients experiencing severe Covid-19.^20^ Furthermore, the UK uses higher thresholds when there is perceived public pressure, as in the case of the Cancer Drugs Fund with an estimated threshold of £220 000/QALY.^29^ The UK’s benefits would outweigh the total GDP loss at above £70 000/QALY (Appendix).

There are possible lessons from our analysis for the ongoing Covid-19 pandemic. One is to consider the cost and QALYs associated with hospitalizations and deaths rather than only total numbers, when making decisions. Successful responses appear to be linked to higher levels of testing or mask wearing rather than strictness of response. The experience of Ireland suggests that consideration should then be given to the maximum potential benefit, which depends on demographics and interpersonal contacts. This final consideration could be applied regionally rather than nationally to better target approaches to future outbreaks.

## Conclusion

Our exploratory analysis estimates that the UK saved 3.17 million (0.32–3.65 million) QALYs, £33 billion (£8–38 billion) in healthcare costs and £1416 (220–1637) HRNB at £20 000/QALY per capita. This is comparable to £1455 GDP loss per capita using Sweden as ‘minimal mitigation’, whereas it is 46.1 (7.1–53.2)% of the total £3075 GDP loss per capita. Germany, Spain and, in most scenarios, Sweden had greater HRNBs per capita and less GDP loss per capita. Ireland fared worst on both measures.

At face value, the total economic impact of Covid-19 exceeded the HRNBs. However, it is not realistic to attribute the full economic impact solely to government responses or to argue that any European country could have avoided their GDP loss or applied ‘no mitigation’. We have attempted to evaluate the extent to which economic costs have been offset by net health benefits. Countries with susceptible populations, higher testing and higher mask wearing, rather than those with the most stringent restrictions, appear to have done better on this measure.

## Data sharing statement

Our model is implemented in the R statistical programming language. Parameters and data are in a Microsoft Excel spreadsheet. Code and data are publicly available: https://github.com/Bogdasayen/covid_cea

## References

[1] WHO. WHO Coronavirus Disease (COVID–19) Dashboard. Available at: https://covid19.who.int/region/euro/country/ (13 November 2020, date last accessed).

[2] IMF. World Economic Outlook Database, Available at: https://www.imf.org/external/pubs/ft/weo/2019/02/weodata/index.aspx (accessed 15 September 2020, date last accessed).

[3] IMF. World Economic Outlook, January 2020: Tentative Stabilization, Sluggish Recovery? Available at: https://www.imf.org/en/Publications/WEO/Issues/2020/01/20/weo-update-january2020 (accessed 1 August 2020, date last accessed).

[4] Scally G , Jacobson B, Abbasi K. The UK’s public health response to covid-19. BMJ 2020;369:m1932. 32414712 10.1136/bmj.m1932

[5] Rowthorn R , Maciejowski J. A cost-benefit analysis of the COVID-19 disease. Oxford Review of Economic Policy 2020;36:S38–S55. 10.1093/oxrep/graa030 PMC7499782 40504226

[6] Miles D, Stedman M, Heald AH. "Stay at Home, Protect the National Health Service, Save Lives": a cost benefit analysis of the lockdown in the United Kingdom. International Journal of Clinical Practice 2020 doi: doi:10.1111/IJCP.13674. 10.1111/ijcp.13674 PMC7435525 32790942

[7] Konig M , Winkler A. COVID-19 and economic growth: does good government performance pay off? Inter Econ 2020;55:224–31. 32834098 10.1007/s10272-020-0906-0 PMC7385207

[8] Chen S , Igan D, Pierri N, Presbitero AF. Tracking the economic impact of COVID-19 and mitigation policies in Europe and the United States. *IMF Working Paper* 2020, WP/20/125.

[9] Zala D, Mosweu I, Critchlow S, et al. Costing the COVID-19 Pandemic: An Exploratory Economic Evaluation of Hypothetical Suppression Policy in the United Kingdom. Value Health 2020;23(11):1432–37. doi: 10.1016/j.jval.2020.07.001al., 10.1016/j.jval.2020.07.001 PMC7833705 33127013

[10] Briggs AH , Goldstein DA, Kirwin E, et al. Estimating (quality-adjusted) life-year losses associated with deaths: With application to COVID-19. Health Econ 2021;30(3):699–707. doi: 10.1002/hec.4208 10.1002/hec.4208 33368853

[11] Davies NG , Wikramaratna PS, Clifford S, et al LSHTM COVID-10 transmission app. Available at: https://cmmid.github.io/visualisations/covid-transmission-model (17 August 2020, date last accessed).

[12] University of Oxford. CORONAVIRUS GOVERNMENT RESPONSE TRACKER. Available at: https://www.bsg.ox.ac.uk/research/research-projects/coronavirus-government-response-tracker (13 November 2020, date last accessed).

[13] Wikipedia. National responses to COVID-19 pandemic. Available at: https://en.wikipedia.org/wiki/National_responses_to_the_COVID-19_pandemic (19 October 2020, date last accessed).

[14] Davies NG , Kucharski AJ, Eggo RM, et al Centre for the mathematical modelling of infectious diseases C-wg. Effects of non-pharmaceutical interventions on COVID-19 cases, deaths, and demand for hospital services in the UK: a modelling study. Lancet Public Health 2020;5:e375–e85. 32502389 10.1016/S2468-2667(20)30133-X PMC7266572

[15] Davies NG , Klepac P, Liu Y, CMMID COVID-19 working group, et al Age-dependent effects in the transmission and control of COVID-19 epidemics. Nat Med 2020;26:1205–11. 32546824 10.1038/s41591-020-0962-9

[16] Flaxman S , Mishra S, Gandy A, Imperial College COVID-19 Response Team, et al Estimating the effects of non-pharmaceutical interventions on COVID-19 in Europe. Nature 2020;584:257–61. 32512579 10.1038/s41586-020-2405-7

[17] Imai N , Cori A, Dorigatti I, et al Report 3: Transmissibility of 2019-nCoV. Available at: https://www.imperial.ac.uk/media/imperial-college/medicine/sph/ide/gida-fellowships/Imperial-College-COVID19-transmissibility-25-01-2020.pdf) (1 October 2020, date last accessed). Imperial College London COVID-19 Response Team 25 January 2020.

[18] Abbott S , Hellewell J, Thompson R, CMMID COVID modelling group, et al Estimating the time-varying reproduction number of SARS-CoV-2 using national and subnational case counts [version 1; peer review: awaiting peer review. Wellcome Open Res 2020;5:112.

[19] PHE. Disparities in the risk and outcomes of COVID-19. Available at: https://assets.publishing.service.gov.uk/government/uploads/system/uploads/attachment_data/file/908434/Disparities_in_the_risk_and_outcomes_of_COVID_August_2020_update.pdf (29 September 2020, date last accessed). Public Health England report, 2020.

[20] National Institute for Health and Care Excellence. Guide to the methods of technology appraisal. *Process and methods guides*, http://publicationsniceorguk/pmg9, 2013. 27905712

[21] Worldometer. Countries in the world by population (2020). Available at: https://www.worldometers.info/world-population/population-by-country/ (3 September 2020, date last accessed).

[22] IMF. World Economic Outlook Update, October 2020: a long and difficult ascent. Available at: https://www.imf.org/en/Publications/WEO/Issues/2020/09/30/world-economic-outlook-october-2020 (7 November 2020, date last accessed).

[23] OECD. Economic Outlook, Volume 2019 Issue 2. Available at: https://www.oecd-ilibrary.org/economics/oecd-economic-outlook-volume-2019-issue-2/summary/english_4c90c873-en (1 August 2020, date last accessed).

[24] OECD. Purchasing power parities (PPP). Available at: https://data.oecd.org/conversion/purchasing-power-parities-ppp.htm (5 October 2020, date last accessed). OECD Data, 2019.

[25] Kulu H , Dorey P. The contribution of age structure to the number of deaths from Covid-19 in the UK by geographical units. *medRxiv* 2020. Available at: 10.1101/2020.04.16.20067991 (22 April 2020, date last accessed).

[26] gov.uk. Policy Paper Spending Review 2020. 25 November 2020. Available at: https://www.gov.uk/government/publications/spending-review-2020-documents/spending-review-2020 (30 November 2020, date last accessed).

[27] Baker R , Bloom N, Davis SJ, Terry SJ. COVID-induced economic uncertainty. Available at: https://www.nber.org/papers/w26983 (1 October 2020, date last accessed). National Bureau of Economic Research, 2020.

[28] Central Statistics Office. National Income and Expenditure 2017: modified gross national income. Available at: https://www.cso.ie/en/releasesandpublications/ep/p-nie/nie2017/mgni/ (19 October 2020, date last accessed).

[29] Leigh S , Granby P. A tale of two thresholds: a framework for prioritization within the Cancer Drugs Fund. Value Health 2016;19:567–76. 27565274 10.1016/j.jval.2016.02.016

